# *Vetufebrus ovatus *n. gen., n. sp. (Haemospororida: Plasmodiidae) vectored by a streblid bat fly (Diptera: Streblidae) in Dominican amber

**DOI:** 10.1186/1756-3305-4-229

**Published:** 2011-12-07

**Authors:** George O Poinar

**Affiliations:** 1 Department of Zoology, Oregon State University, Corvallis, OR 97331, USA

**Keywords:** Dominican Republic amber, Fossil bat malaria, *Vetufebrus ovatus*, n. gen., n. sp

## Abstract

**Background:**

Both sexes of bat flies in the families Nycteribiidae and Streblidae (Diptera: Hippoboscoidea) reside in the hair or on the wing membranes of bats and feed on blood. Members of the Nycteribiidae transmit bat malaria globally however extant streblids have never been implemented as vectors of bat malaria. The present study shows that during the Tertiary, streblids also were vectors of bat malaria.

**Results:**

A new haemospororidan, *Vetufebrus ovatus*, n. gen., n. sp., (Haemospororida: Plasmodiidae) is described from two oocysts attached to the midgut wall and sporozoites in salivary glands and ducts of a fossil bat fly (Diptera: Streblidae) in Dominican amber. The new genus is characterized by ovoid oocysts, short, stubby sporozoites with rounded ends and its occurrence in a fossil streblid. This is the first haemosporidian reported from a streblid bat fly and shows that representatives of the Hippoboscoidea were vectoring bat malaria in the New World by the mid-Tertiary.

**Conclusions:**

This report is the first evidence of an extant or extinct streblid bat fly transmitting malaria. Discovering a mid-tertiary malarial parasite in a fossil streblid that closely resembles members of a malarial genus found in nycteribiid bat flies today shows how little we know about the vector associations of streblids. While no malaria parasites have been found in extant streblids, they probably occur and it is possible that streblids were the earliest lineage of flies that transmitted bat malaria to Chiroptera.

## Background

Amber is known for its ability to preserve vertebrate microbial pathogens. Thus far, there are records of malaria, leishmaniasis and trypanosomiasis associated with insect vectors in amber deposits ranging from 20 to 100 million years of age [1]. Sporogonic stages of the bird malaria, *Plasmodium dominicana*, occurred in *Culex malariager *in Dominican amber [2] and developmental stages of *Paleohaemoproteus burmacis *(Haemospororida: Plasmodiidae) were reported from an Early Cretaceous Burmese amber biting midge (Diptera: Ceratopogonidae) [3]. The present study describes the sporogonic stages of a new genus of bat malaria in a Dominican amber fossil streblid [4]. Extant streblids have never been implicated as vectors of bat malaria, however members of the closely related family Nycteribiidae transmit bat malaria globally [5,6] (Table 1). Since both sexes of streblid bat flies (Diptera: Hippoboscoidea: Streblidae) feed on the blood of bats and reside in the hair or on the wing membranes [7-9], they would be excellent vectors of bat malaria.

**Table 1:** Hippoboscoidea vectors of malaria

Vector	Malaria type	Vertebrate	Reference
Hippoboscidae			
*Icosta hirsuta*	*Haemoproteus lophortyx*	birds	[8]
*I. rufiventris*	*H. lophortyx*	birds	[8]
*Lynchia hirsuta*	*H. lophortyx*	birds	[20]
*Microlynchia pusilla*	*H. columbae*	birds	[21]
*M. pusulla*	*H. maccallumi*	birds	[22]
*M. pusilla*	*H. sacherovi*	birds	[22]
*Ornithomyia avicularia*	*H. mansoni*	birds	[23]
*Ornithomyia lagopodia*	*H. mansoni*	birds	[24]
*Pseudolynchia brunnea*	*H. columbae*	birds	[21]
*P. canariensis*	*H. columbae*	birds	[25]
*P. canariensis*	*H. maccallumi*	birds	[22]
*P. capensis*	*H. columbae*	birds	[21]
*P. maura*	*H. columbae*	birds	[21]
*Stilbometopa impressa*	*H. lophortyx*	birds	[26]
Nycteribiidae			
*Basilia *sp.	*Polychromophilus deanei*	bats	[12]
*Listropoda *sp.	?	bats	[27]
*Nycteribia dentata*	*Polychromophilus *sp.	bats	[28]
*N. kolenatii*	*P. murinus*	bats	[10]
*N. parvula*	*P. melanipherus*	bats	[29]
*Penicillidia fulvida*	*Polychromophilus *sp.	bats	[11]
*Penicillidia *spp.	*Polychromophilus *sp.	bats	[13]

## Methods

### Specimen

**Figure 1:**
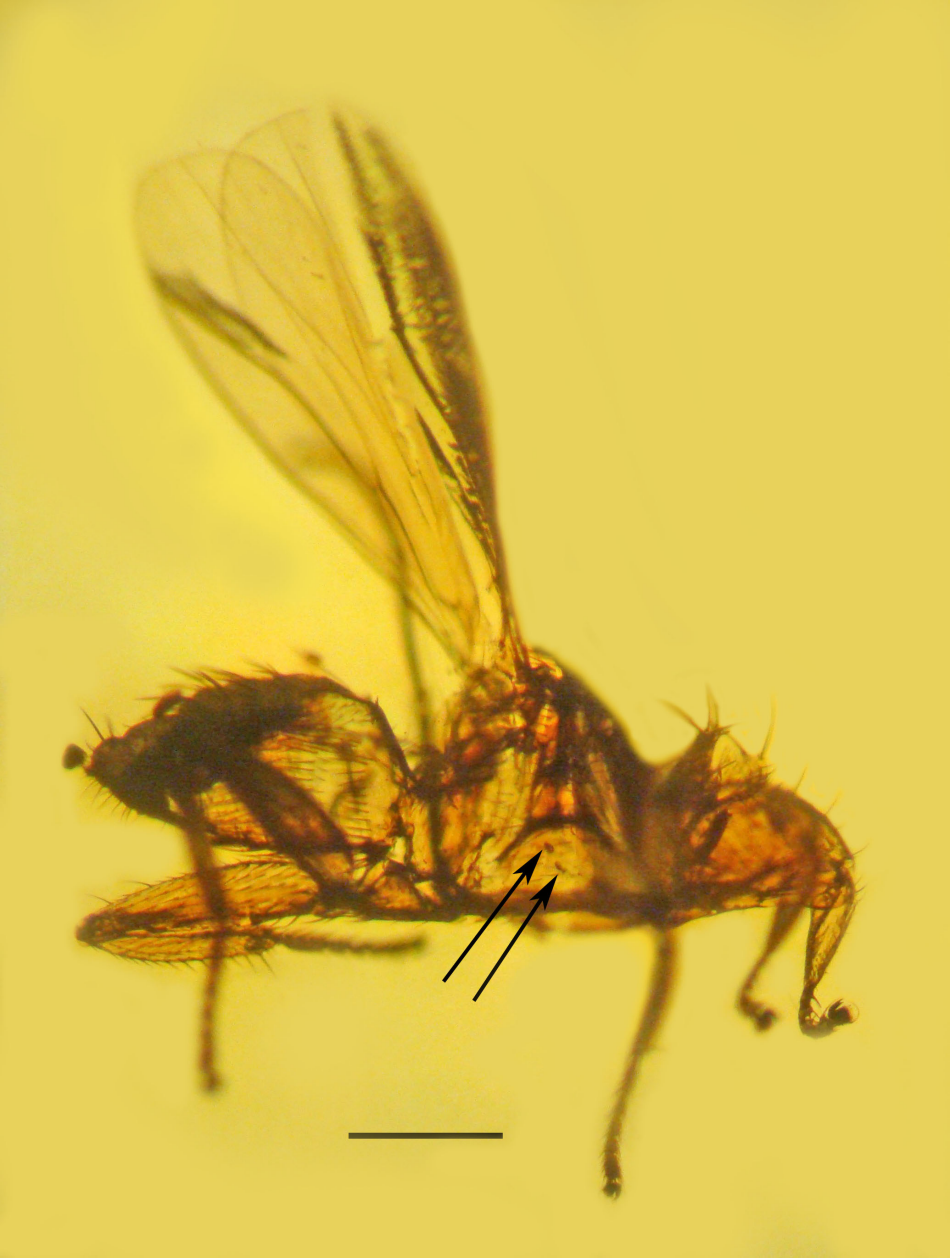
**Two oocysts**. Two oocysts (arrows) of *Vetufebrus ovatus *n. gen., n. sp. on the gut wall of a Dominican amber streblid bat fly. Bar = 272 μm.

The fossil streblid is in a piece of amber 5 mm long by 4 mm wide by 4 mm deep (Figure 1). Observations, drawings, and photographs were made with a Nikon SMZ-10 R stereoscopic microscope and Nikon Optiphot compound microscope with magnifications up to 600×.

Since it was not possible to photograph the malarial organisms without polishing away portions of the fly, photographs had to be taken through the thickness of the amber matrix as well as the width of the body wall of the vector. Adobe Photoshop was used to enlarge the photos and obtain the clearest images.

### Locality

The amber with the bat fly came from La Búcara mine in the Cordillera Septentrional of the Dominican Republic. Dating of Dominican amber is still controversial with the latest purposed age of 20-15 mya based on foraminifera [10] and the earliest as 45-30 mya based on coccoliths [11]. In addition, Dominican amber is secondarily deposited in sedimentary rocks, which makes a definite age determination difficult [12]. A range of ages for Dominican amber is possible since the amber is associated with turbiditic sandstones of the Upper Eocene to Lower Miocene Mamey Group [13]. Dominican amber was produced by the leguminous tree, *Hymenaea protera *Poinar [14] and a re-construction of the Dominican amber forest based on amber fossils indicated that the environment was similar to that of a present day tropical moist forest [15].

## Results and Discussion

### Description of malarial pathogen

Phylum Apicomplexa Levine, 1970

Class Aconoidasida Mehlhorn, Peters & Haberkorn, 1980

Order Haemospororida Danilewsky, 1885

The description is based on two oocysts and sporozoites in the oocysts and salivary glands/secretions of a fossil streblid bat fly [4].

**Figure 2:**
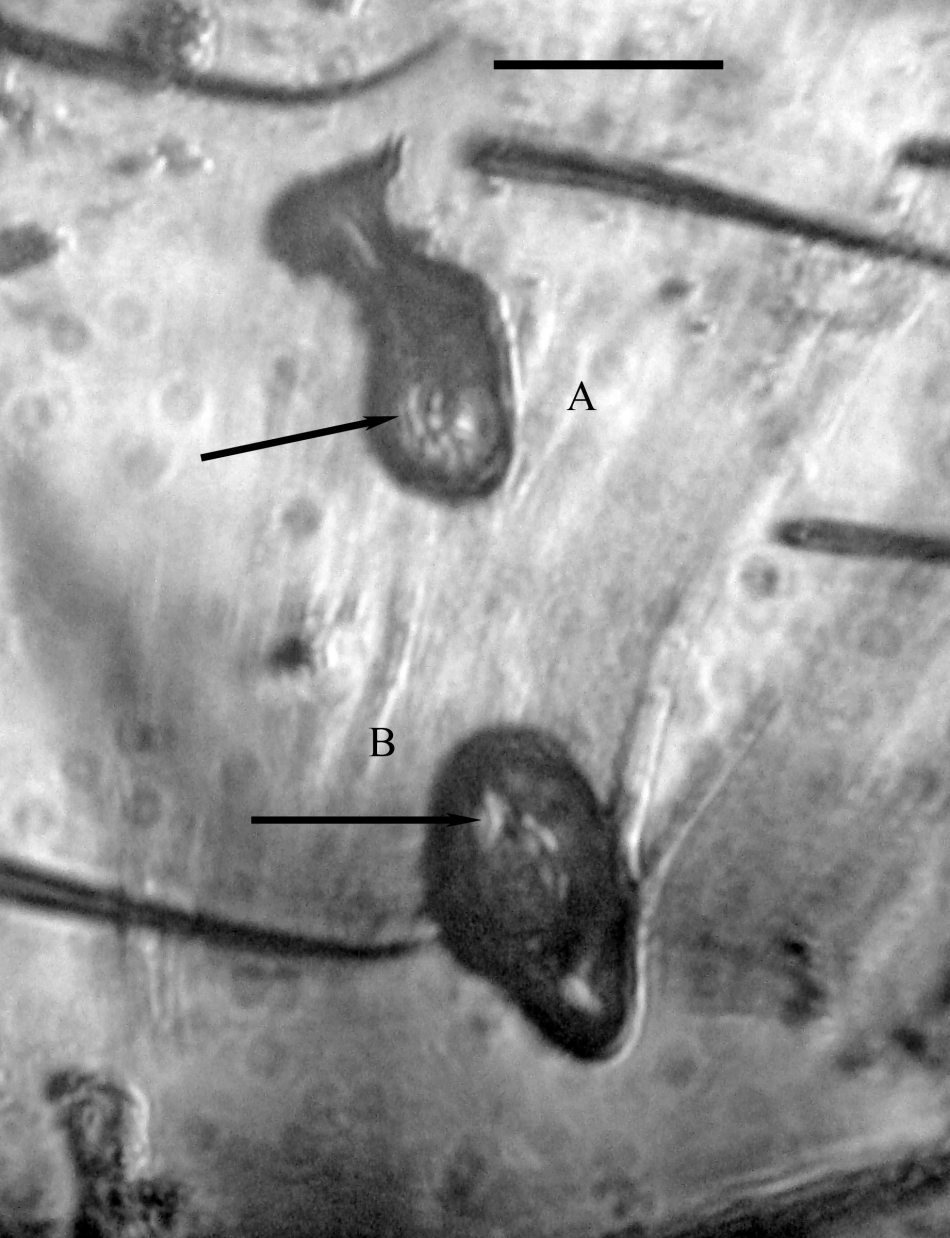
**Oocysts A and B of *Vetufebrus ovatus***. Oocysts A and B of *Vetufebrus ovatus *n. gen., n. sp. attached to the gut wall of a Dominican amber streblid bat fly. Arrows show developing sporozoites inside oocysts. Bar = 33 μm.

**Figure 3:**
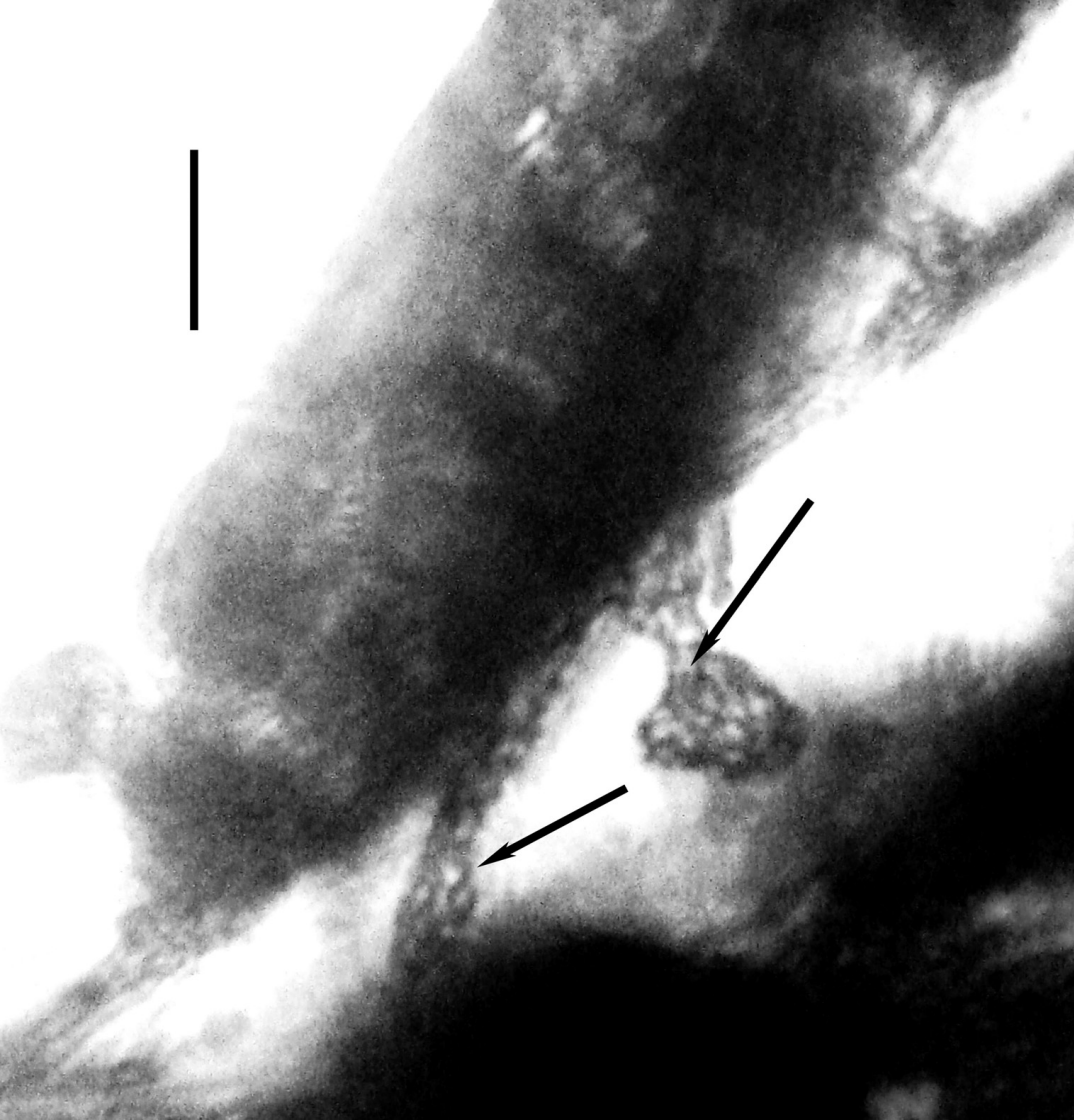
**Sporozoites (arrows) of *Vetufebrus ovatus***. Sporozoites (arrows) of *Vetufebrus ovatus *n. gen., n. sp. in salivary glands and ducts of a Dominican amber streblid bat fly. Bar = 20 μm.

**Figure 4:**
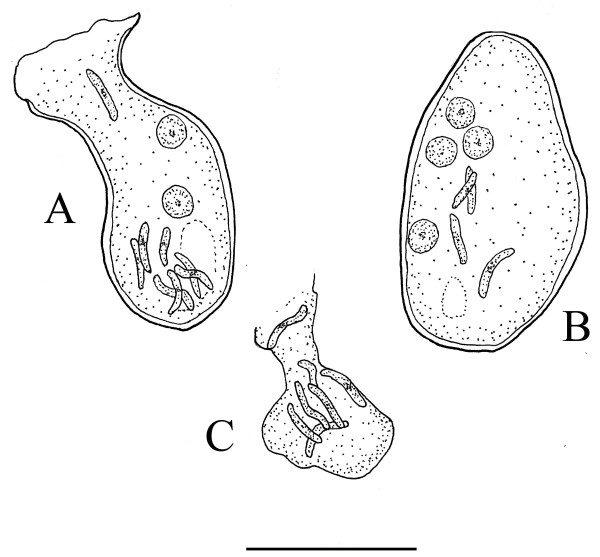
**Oocysts of *Vetufebrus ovatus***. A and B. Oocysts of *Vetufebrus ovatus *n. gen., n. sp. containing nucleated cells and developing sporozoites in a Dominican amber streblid bat fly. C. Sporozoites of *Vetufebrus ovatus *n. gen., n. sp. in a salivary gland secretion of a Dominican amber streblid bat fly. Bar = 27 μm.

*Vetufebrus *Poinar n. gen. (Figures 1, 2, 3 and 4)

Description. Oocysts small, oval, with nucleated cells 3-5 μm in diameter and developing sporozoites 7-10 μm in length; sporozoites in salivary glands and ducts stubby, with rounded ends, 8-10 μm in length; occurs in a Dominican amber streblid bat fly [4].

Type species: *Vetufebrus ovatus *Poinar

*Vetufebrus ovatus *Poinar, n. sp.

Description: Oocysts brown; oocyst A, 32 μm × 15 μm; surrounding membrane 1.2 -1.4 μm wide, containing dark nucleated cells 3-5 μm in diameter and developing sporozoites 7-10 μm in length; oocyst B, 29 μm × 17 μm, containing dark nucleated cells 3-5 μm in diameter and developing sporozoites 7-10 μm in length; surrounding membrane 1.2-1.4 μm wide; sporozoites in salivary glands/secretions short, stubby, with rounded ends, 8-10 μm in length.

Etymology: The generic name is from the Latin "vetus" for old and the Latin "febris" for fever. The specific epithet is from the Latin "ovatus" for ovate, referring to the shape of the oocysts.

Holotype. Specimen (accession # D-7-239) deposited in the Poinar amber collection maintained at Oregon State University, Corvallis, Oregon.

Locality: La Búcara amber mine (19°13' × 70°40') in the northern portion of the Dominican Republic.

## Conclusions

The present study represents the first description of a haemosporidian reported from a streblid bat fly and shows that representatives of the Hippoboscoidea were vectoring bat malaria in the mid-Tertiary. The presence of sporozoites in salivary glands and ducts indicates that the streblid was a successful vector of *Vetufebrus*.

Bats are infected with four genera of malaria: *Plasmodium*, *Hepatocystis, Nycteria *and *Polychromophilus *[5,6]. Species of the former three genera infect Old World bats while *Polychromophilus *occurs globally and is the only bat malaria reported from the New World [16]. All known strains of *Polychromophilus *are vectored by nycteriibid flies of the genera *Nycteribia, Penicillidia *and *Basilia *(Table 2).

**Table 2:** Sporogonic stages of bat malaria in Hippoboscoidea (all Nycteriibidae except for *Vetufebrus *in a fossil streblid).

Malaria	Vector	Oocyst	Sporozoite	Reference
*Vetufebrus ovatus *	Fossil streblid	35 × 15,29 × 17 oval	7-10, stubby ends rounded	Present study
*P. deanei*	*Basilia *sp.	60, round	8, thick	[12]
*P. melanipherus*	*Nycteribia parvula*	?	?	[29]
*P. murinus*	*Nycteribia kolenatii*	57-71, round	7.4	[10]
*Polychromophilus sp*.	*Nycteribia dentata*	?	7, stubby ends rounded	[28]
*Polychromophilus sp*.	*Penicillidia fulvida*	oval, 31 × 10 57 × 47	13, thick, blunt ends	[11]
*Polychromophilus *sp.	*P. dufouri* *P. conspicua*	?	stubby, blunt ends	[5,13]
*Polychromophilus *?	*Listropoda *sp.	?	6.3, stubby, blunt ends	[5,27]

All measurements in microns.

The sporogonic stages of *Polychromophilus *are characterized by round, slow growing oocysts attached to the midgut of the vector and short, stubby sporozoites with rounded ends [5]. The mature round oocysts of *Polychromophilus *spp. contrast with the small oval oocysts of *Vetufebrus *(Table 2)(Figures 2, 4A, B). However, Adam and Landau [17] noted a small (31 × 10 μm), oval *Polychromophilus *oocyst in the nycteribiid fly, *Penicillidia fulvida *Bigot, 1889. It is likely that this oval oocyst in the nycteribiid was still developing, which could be the case with the two oocysts of *Vetufebrus*. While the sporozoites noted in the two oocysts of *Vetufebrus *are similar in size and shape to those in the salivary glands, it is possible that some of the salivary gland sporozoites originated from an earlier infection.

Short and stubby sporozoites with rounded ends as reported here for *Vetufebrus *(Figures 3, 4C) are characteristic of *Polychromophilus *infections [17-19](Table 2) and the dimensions of *Vetufebrus *sporozoites fall within the range of some *Polychromophilus *spp. (Table 2). While *Vetufebrus *could represent an early lineage of *Polychromophilus*, this is unclear due to the small size of the oocysts. Also bat malaria has not been found in extant streblids even though species of the closely related Nycteribiidae carry bat malaria (Table 1). Adam & Landau [17] found no malarial stages in the streblids, *Raymondia simplex *Jobling 1954, *R. seminuda *Jobling, 1954 and *R. leleupi *Jobling 1954 while searching for vectors of *Polychromophilus *in the Congo Republic. Also, Garnham [5] found no sporogonic stages of malaria in African streblids.

In accordance with section 8.6 of the ICZN's International Code of Zoological

Nomenclature, copies of this article are deposited at the following five publicly accessible libraries: Natural History Museum, London, UK; American Museum of Natural History, New York, USA; Museum National d'Histoire Naturelle, Paris, France; Russian Academy of Sciences, Moscow, Russia; Academia Sinica, Taipei, Taiwan.

## Competing interests

The author declares that they have no competing interests.

## Authors' contributions

GP discovered the haemosporidian, designed and wrote the paper and supplied the figures.
